# Supplementary material for: Malignant Neoplasms Arising in the Cardiac Pacemaker Cavity: A Systematic Review
Source: Cancers (Basel). 2023 Oct 29;15(21):5206. doi: 10.3390/cancers15215206 (PMC10647525; doi:10.3390/cancers15215206)
Supplement: Supplementary file 1 [file cancers-15-05206-s001.zip › cancers-2615369-supplementary.pdf]

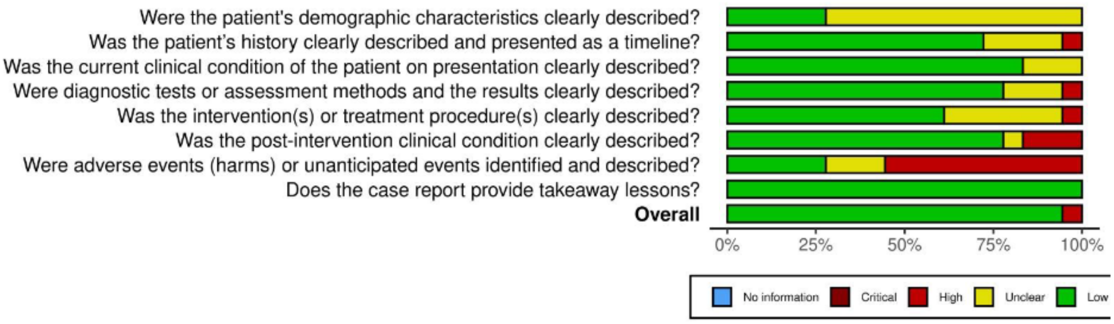

Figure S1. Graphical representation regarding "Figure 2: Risk of bias among case reports".

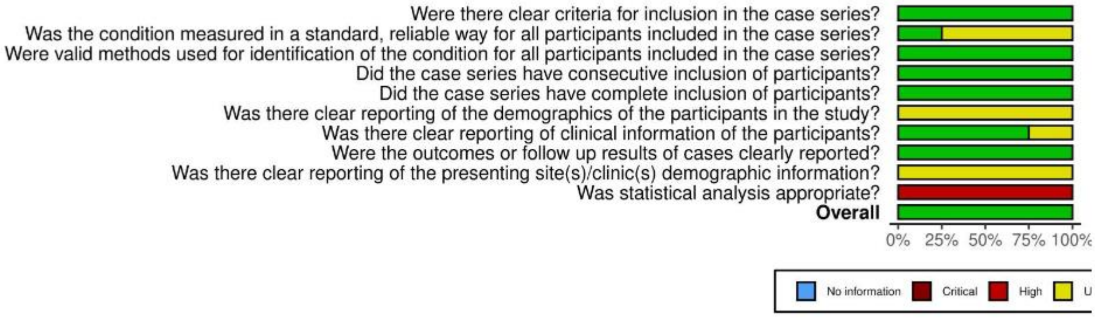

Figure S2. Graphical representation regarding "Figure 3: Risk of bias among case series".

Table S1. Tests used, conduct and clinical outcomes.

AUTHOR	LAB EXAMS	ECHO	ECG	CT	MG	USG	RADIOGRAPHY	BIOPSY	MANAGEMENT	OUTCOME
						**Case Reports**				
De Mattia; Brieda; Dametto (2011) [36]	NA	NA	NA	NA	Round spiculated mass caudal to the pacemaker pocket	Subcutaneous solid mass containing calcium deposits	NA	Invasive Ductal Carcinoma	NA	NA
Fraedrich et al. (1984) [37]	NA	NA	NA	NA	NA	NA	Shadow in the posterobasal segment of the left lung suspected of peripheral bronchial carcinoma with enlargement	Malignant fibrous histiocytoma	Removal of tumor mass, plastically closed defect after pacemaker placement more laterally	Death due to cachexia
González-Vela et al. (2009) [30]	NA	NA	NA	NA	NA	NA	NA	Ill-defined lesion with two different patterns: An area composed of fascicular interweaving of spindle cells with rounded nuclei, mild nuclear pleomorphism, and scant mitosis (1 mitosis per 10 high-power fields). The other area showed large spindle-shaped cells with moderate nuclei, pleomorphism and rapid mitotic activity (35 mitoses per 10 high power fields). Diagnosis of cutaneous leiomyosarcoma	Surgical excision for histopathological study with new resection to expand the excision margins.	12 months after surgery the patient was well and disease free.

González-Vela et al. (2013) [31]	NA	NA	NA	NA	NA	NA	NA	Dermal proliferation with bizarre fusiform and polygonal cells and atypical mitosis, severe pleomorphism and hyperchromatism, ulcerated epidermis, areas of intratumoral necrosis constituting 10% of the neoplasm with minimal subcutaneous involvement, free surgical borders, spindle cells with expression of CD10, CD99, CD68 (focal) and smooth muscle actin (focal), S100 protein, Melan-A, desmin, CD34, p63, CD31 and human herpesvirus latent nuclear antigen 8 8. Diagnosis of Atypical Fibroxanthoma.	Cross section revealed hard, dense, gray and white tumor next to deep surgical margin, new surgical resection to widen excision margins	Absence of recurrent disease 9 months after surgery
Hamaker et al. (1976) [24]	Hypercalcemia, hyperuricemia, uremia, anemia, leukopenia and thrombocytopenia	NA	AV block with functional pacemaker	NA	NA	NA	Transvenous pacemaker with soft tissue mass around the pulse generator in the right pectoral area	Bone marrow sample with 20% plasma cells, bone scintigraphy with increased uptake area on the posterior side of the ribs in the region of the right scapula	Contact with pacemaker manufacturer, cobalt therapy with 2000 rads at pacemaker site, reduction in tumor volume, dialysis, nitrogen mustard therapy.	Death from progressive multiple myeloma, and sepsis after maximal supportive effort.
Herrmann; Mishra; Greenway, (2014) [25]	NA	NA	Ventricular rhythm at 60 bpm in pre, intra and postoperative	NA	NA	NA	NA	Nodular and Superficial Basal Cell Carcinoma transected in the deep margin.	Mohs micrographic surgery.	At 7 weeks of follow-up the scar was healing properly.

			periods							
Hojo et al. (2003) [43]	Elevated soluble interleukin 2 receptor, normal lactate dehydrogenase	NA	Mobitz II AV block with 4.2-second ventricular asystole	NA	NA	NA	NA	Diffuse large B-cell lymphoma	30 Gy of radiotherapy to the upper left side of the chest and left armpit	Patient stable and cured 16 months after treatment
Knez et al. (1999) [39]	NA	NA	NA	NA	NA	NA	NA	Poorly differentiated invasive ductal carcinoma. Multiple foci of intraductal carcinoma. Other foci of ductal carcinoma in situ revealed uniform clear cells with a distinct growth pattern and cell margins, focally showing signs of intraluminal necrosis.	Excision of the necrotic and pyogenic tissue surrounding the lesion, complete removal of the Pacemaker pocket, modified radical mastectomy with axillary lymph node dissection..	At the 20-month follow-up, the patient remained free of metastasis and disease.
Khamooshian et al. (2017) [41]	ND	Left ventricular ejection fraction reduced by 35%	ND	30 mm round nodule in left shoulder without pulmonary metastasis or mediastinal/hilar lymphadenopathy	ND	Lobulated, hypoechogenic, solid lesion measuring 23 x 15 x 78 mm with well-defined borders surrounding the pacemaker leads	ND	Pleomorphic malignant cells with hyperchromatic and polymorphic nuclei, tumor-free margins	VVI-R single-chamber pacemaker in the rectus abdominis muscle with extension to the epicardial electrode of the left ventricle; radical resection of the tumor, old electrodes, deltoid and pectoralis major through a 12x8 cm horseshoetype caudal incision containing a skin flap for reconstruction.	Intermediate ejection fraction of 45%, hospital discharge on the 3rd postoperative day, adjuvant radiotherapy of 60 Gy
Magilligan, Isshak (1980) [26]	ND	ND	Complete atrioventricular dissociation without pacemaker activity	ND	ND	ND	Satisfactory lead position in the right ventricle, with no apparent lead fracture	Infiltrating adenocarcinoma in the pacemaker pocket and not extending to the previous mastectomy incision	Insertion of transvenous pacemaker via left cephalic vein with subcutaneous implantation of generator over left pectoralis major muscle, thoracic mass excised widely	Patient recovered uneventfully

Milner et al.(2021) [29]	Mild normochromic and normocytic anemia, QuantiFERON-TB Gold positive	Intermediate grade left ventricular ejection fraction, without infective endocarditis	ND	Soft tissue mass over the pacemaker generator measuring 65 by 24 mm, in contact with the pectoralis major muscle in the deep plane, in addition to several left axillary lymphadenopathies, the largest of which measuring 10 mm	ND	ND	No visible changes	Massive infiltration of the entire dermis by a neoplasm with plasmacytic differentiation, with positivity for CD138 and CD4 and kappa light chain restriction, Ki-67 proliferative index close to 90%, allowing primary cutaneous plasmacytoma or cutaneous infiltration by multiple myeloma. His bone marrow aspiration and biopsy were compatible with the diagnosis of lymphoplasmacytic lymphoma with bone marrow infiltration.	First entry: Drainage of the hematoma in the region of the pacemaker pocket, oral flucloxacillin; Second hospitalization: watchful waiting due to the spontaneous regression of the neoplastic lesion, referral to the tuberculosis clinic, isoniazid and pyridoxine	Spontaneous regression of the skin lesion without the need for chemotherapy treatment, persistent mild anemia, referral to the tuberculosis outpatient clinic and isoniazid and pyridoxine prescribed, outpatient follow-up without complaints reported by the patient
Moruzzo et al. (2009) [35]	Lactate dehydrogenase: 496 U/L, small amount of M protein (lambda Ig-A)	ND	ND	Mass 6 cm in diameter, coliquative nucleus in the RVDR pacemaker pocket, diffuse thoracic lymphadenopathy with a maximum diameter of 1.2 cm	ND	ND	ND	Large cell lymphoma, null cell immunophenotype, CD3, CD20, CD79a negative, CD138 and CD30 positive and CD43 positive in a focal form indicating plasmablastic undifferentiation, classified as Non-Hodgkin's Lymphoma stage IE, B, IPI 2 (low intermediate risk)	Chemotherapy with cyclophosphamide, doxorubicin, vincristine, prednisone and was discharged, chemotherapy in the hospital near his hometown	Death after pneumonia during post-therapy neutropenia
Rasmussen et al. (1985) [34]	ND	ND	ND	ND	ND	ND	ND	Papillary adenocarcinoma infiltrating the fibrous wall of the pacemaker pocket, tumor metastases in the axillary lymph nodes	Removal of right breast with pacemaker bag and abnormal right armpit lymph nodes, radiation and anti-estrogen drugs	Death 2 months later due to generalized metastases

Rathinam et al. (2010) [32]	Hb: 6.1g/L ESR: 35mm/h, CRP: 155mg/L Blood culture: negative for growth of organisms.	No evidence of endocarditis	ND	8-9cm round mass in the left clavicular region superficial to the left pectoral muscle, on the upper surface of the pacemaker	ND	ND	ND	Compact fascicles of spindle cell proliferation with prominent inflammatory component composed of lymphocytes, plasma cells and eosinophils. Numerous foci of necrosis. Spindle cells reacted strongly and diffusely with CD-34, but were negative for pancytokeratin, smooth muscle actin, desmin, Protein S100, anaplastic lymphoma Kinase. Diagnosis of Inflammatory Myofibroblastic Tumor.	Placement of a new Pacemaker in the right (opposite) infraclavicular region. Removal of the mass together with the pacemaker performed en-bloc, with wide margin resection. Pasta was well encapsulated to the pacemaker and its wires. Patient was discussed in multidisciplinary meetings and adjuvant local radiotherapy was decided.	Postoperative uneventful. Constitutional symptoms disappeared. Laboratory returned to normal. Appetite and weight returned.
Reyes (2008) [27]	ND	ND	ND	ND	No abnormalities or suspicious changes in either breast	Lesion of the mass near the pacemaker	ND	Malignant epithelial cells, clear cell hidradenocarcinoma, metastatic lobular carcinoma in the right lateral thoracic skin was confirmed by positive estrogen receptor, progesterone receptor, mammaglobin and cytokeratin CK7	Recommended irradiation of the operative area and right breast was refused by the patient, arimidex in symptomatic and supportive care, 2 subsequent annual mammograms normal and negative for suspicious lesion	Complete autopsy revealed no recurrent tumor in the right lateral pectoral area, primary breast malignancy, or other metastatic lesion, confirming diagnosis of hidradenocarcinoma

Rothenberger-Janzen; Flueckiger; Bigler (1998) [42]	ND	ND	ND	No evidence of regional or distant metastasis	ND	ND	No evidence of regional or distant metastasis	Wide excision of the tumor revealed intraductal adenocarcinoma with extracellular mucus. TNM Classification: pT2, G2	Wide excision of the tumor with adjuvant Tamoxifen chemotherapy	After 7 months he is well and disease free
Zarifi et al.(2017) [28]	White blood cells 12.8x10 9/L, erythrocyte sedimentation rate 56 IU/L, procalcitonin 0.18 ng/mL, high sensitivity PCR 7.64 mg/dL, troponin 0.032 ng/mL, CKMB 5.6 ng/mL	ND	ND	Absence of fluid collection	ND	Complex fluid adjacent to the pacemaker compatible with hematoma versus abscess	ND	Plasmacytic lymphoblasts with abundant blue cytoplasm, large nuclei and paranuclear hof, positive immunohistochemical staining for Ki-67 and CD-138	Intravenous piperacycline/tazobactam for proximal abscess, cardiac catheterization for device removal, cefazolin for bag irrigation	Non-pacemaker dependent patient chose not to implant another device, palliative care, without chemotherapy
Zonca et al. (2009) [40]	NA	NA	NA	NA	Findings difficult to interpret	Suspicious lesion in the pacemaker pocket	ND	Invasive ductal carcinoma	Radiotherapy and chemotherapy with tamoxifen	Death 25 months after tumor diagnosis
**Case series**										
Bhandarkar; Bewu; Taylor, (1993) [33]	Biochemical parameters within reference values	ND	ND	ND	ND	ND	Pacemaker lying on the ribcage on the left side, corresponding to the clinically palpable mass	Adenocarcinoma (unspecified)	Due to his advanced age, we opted for anti-estrogenic chemotherapy with Tamoxifen	ND
	ND	ND	ND	ND	ND	ND	ND	Adenocarcinoma (unspecified)	Due to age and poor general condition, treatment with tamoxifen was chosen.	ND

Biran et al. (1979) [44]	ND	ND	Alternating second- and third-degree AV block	ND	ND	ND	ND	intraductal carcinoma	Right radical mastectomy. Cytoxan, Methotrexate and 5-fluoracil chemotherapy.	ND
	ND	ND	ND	ND	ND	ND	ND	Adenocarcinoma and Paget's disease with normal axillary lymph nodes	Pacemaker transfer to the left. Radical mastectomy with right axillary lymphadenectomy. Adjuvant radiotherapy to the right pectoral	2 years after the mastectomy the patient feels well.
Moseley et al. (2021) [19]	No laboratory abnormalities	No evidence of endocarditis.	ND	ND	ND	ND	ND	Recurrence of large B-cell lymphoma.	Debridement and capsulectomy of the original pacemaker with placement of a new pacemaker in the left subpectoral region. Released for 3 weeks on an oral doxycycline regimen. There was worsening of the surgical site with hardening and exposure of vascularized tissue. New exploration of the pouch, debridement and closure of the primary wound was performed.	ND
	Positive wound culture for Staphylococcus aureus, Enterococcus faecalis, and Pseudomonas aeruginosa.	Negative for endocarditis	ND	ND	ND	ND	ND	Biopsy of the lesion revealed moderately differentiated squamous cell carcinoma extending into the deep margin and focally into the peripheral margins. Arm lump biopsy showed B-cell lymphoma	Removal of pulse generator and extraction of 3 permanent transvenous pacemaker leads. After 72 hours of negative blood cultures, a pacemaker without electrodes (Medtronic Micra) was placed. Cleared with a 2-week	He is currently being followed up by an oncologist for Squamous Cell Carcinoma and B-Cell Lymphoma

	Negative blood cultures.								regimen of intravenous piperacillin/tazobactam antibiotics. Later infusions were used to treat the lymphoma.	
Zafirocopoulos; Rouskas, (1974) [38]	ND	ND	ND	Nodular bilateral round shadows typical of metastatic lesions in lungs	ND	ND	Nodular bilateral round shadows typical of metastatic lesions in lungs. osteolytic lesion in the skull	Axillary nodule with infiltration of Schirrous Adenocarcinoma	Treatment with cytotoxic drugs (unspecified)	Death 5 months after initiation of treatment
	ND	ND	ND	ND	ND	ND	ND	Excision of the sinus showed Schirroous Adenocarcinoma extending to the anterior Pacemaker Pouch scar. No signs of metastatic lesions elsewhere.	Simple mastectomy with supplemental cobalt therapy.	Eight months after the mastectomy, the patient is well and free of metastases.

Abbreviations: NA: non-available; AV: atrioventricular; bpm: beats per minute; rads: radiation unit equal to 0.01 of a Gy unit; Gy: gray; CRP: C-Reactive Protein; CKMB: Creatine Phosphokinase MB; Hb: Hemoglobin; ND: Not described; AVB: total atrioventricular block; AVB: atrioventricular block; BPM: beats per minute; Gy: Gray; ECHO: Echocardiogram; ECG: Electrocardiogram. USG: Ultrassonography; MG: Mammography; CT: Computerized Tomography
